# Non-traumatic osteonecrosis of the femoral head induced by steroid and alcohol exposure is associated with intestinal flora alterations and metabolomic profiles

**DOI:** 10.1186/s13018-024-04713-z

**Published:** 2024-04-12

**Authors:** Qing-Yuan Zheng, Ye Tao, Lei Geng, Peng Ren, Ming Ni, Guo-Qiang Zhang

**Affiliations:** 1 Medical School of Chinese PLA, Beijing, 100853 China; 2 Department of Orthopedics, the First Medical Center, Chinese People’s Liberation Army General Hospital, Fuxing Road, Haidian District, Beijing, 100853 China; 3 Department of Orthopedics, the Fourth Medical Center, Chinese PLA General Hospital, Beijing, 100853 China

**Keywords:** Non-traumatic femoral head necrosis, Intestinal flora, Metabolomic profiles, Steroid, Alcohol

## Abstract

**Objective:**

Osteonecrosis of the femoral head (ONFH) is a severe disease that primarily affects the middle-aged population, imposing a significant economic and social burden. Recent research has linked the progression of non-traumatic osteonecrosis of the femoral head (NONFH) to the composition of the gut microbiota. Steroids and alcohol are considered major contributing factors. However, the relationship between NONFH caused by two etiologies and the microbiota remains unclear. In this study, we examined the gut microbiota and fecal metabolic phenotypes of two groups of patients, and analyzed potential differences in the pathogenic mechanisms from both the microbial and metabolic perspectives.

**Methods:**

Utilizing fecal samples from 68 NONFH patients (32 steroid-induced, 36 alcohol-induced), high-throughput 16 S rDNA sequencing and liquid chromatography with tandem mass spectrometry (LC-MS/MS) metabolomics analyses were conducted. Univariate and multivariate analyses were applied to the omics data, employing linear discriminant analysis effect size to identify potential biomarkers. Additionally, functional annotation of differential metabolites and associated pathways was performed using the Kyoto Encyclopedia of Genes and Genomes (KEGG) database. Subsequently, Spearman correlation analysis was employed to assess the potential correlations between differential gut microbiota and metabolites.

**Results:**

High-throughput 16 S rDNA sequencing revealed significant gut microbial differences. At the genus level, the alcohol group had higher *Lactobacillus* and *Roseburia*, while the steroid group had more *Megasphaera* and *Akkermansia*. LC-MS/MS metabolomic analysis indicates significant differences in fecal metabolites between steroid- and alcohol-induced ONFH patients. Alcohol-induced ONFH (AONFH) showed elevated levels of L-Lysine and Oxoglutaric acid, while steroid-induced ONFH(SONFH) had increased Gluconic acid and Phosphoric acid. KEGG annotation revealed 10 pathways with metabolite differences between AONFH and SONFH patients. Correlation analysis revealed the association between differential gut flora and differential metabolites.

**Conclusions:**

Our results suggest that hormones and alcohol can induce changes in the gut microbiota, leading to alterations in fecal metabolites. These changes, driven by different pathways, contribute to the progression of the disease. The study opens new research directions for understanding the pathogenic mechanisms of hormone- or alcohol-induced NONFH, suggesting that differentiated preventive and therapeutic approaches may be needed for NONFH caused by different triggers.

**Supplementary Information:**

The online version contains supplementary material available at 10.1186/s13018-024-04713-z.

## Introduction

Osteonecrosis of the femoral head (ONFH) is a severe medical condition that primarily affects young to middle-aged adults [1]. In its advanced stages, ONFH can lead to femoral head collapse, necessitating total hip arthroplasty (THA) [2, 3]. According to projections by Mont et al., the global total of ONFH patients is expected to reach 20 million over the next decade [4]. In the United States, the annual incidence of ONFH is estimated to be over 20,000 cases [2]. It is estimated that approximately 10 million people in China are affected by ONFH. ONFH frequently results in severe functional impairments and can even lead to disability, imposing significant economic burdens on both patients and their families, as well as on society at large [5].

However, there is no clear consensus regarding the specific mechanisms and causes underlying the occurrence and development of ONFH. The primary causes of ONFH include alcohol consumption (37.15%), steroid use (26.84%), and trauma (15.73%), accounting for nearly 80% of cases associated with these three factors [6, 7]. The pathogenesis of traumatic ONFH is relatively well understood, with mechanical trauma being a major contributor to impairment of blood supply to the femoral head [8]. Hines et al. suggested that alcohol-induced ONFH (AONFH) and steroid-induced ONFH (SONFH) may involve distinct mechanisms in their pathogenesis [9].

The potential mechanisms underlying SONFH include disturbances in lipid metabolism, reduced osteogenic potential, compromised blood supply, inflammation, cell apoptosis, and genetic factors [10, 11]. Liu et al. analyzed the plasma metabolomic profile of ONFH patients, revealing significant alterations in the expression levels of lipids, glutathione, nucleotides, and energy-related metabolites. These metabolites are associated with disruptions in lipid and energy metabolism, as well as heightened oxidative stress, suggesting that disturbances in energy metabolism and oxidative stress might be potential mechanisms contributing to the development of ONFH [12].

Furthermore, some preliminary animal experimental studies have indicated that the interplay between GM composition and its metabolites significantly contributes to the onset and progression of ONFH [8]. This is an intriguing avenue of investigation, as the impact of alcohol and steroids on the GM is well established [13, 14], and both alcohol and steroids are recognized as major contributors to the induction of non-traumatic ONFH. There may be meaningful correlations within this context. Although research in this area is still limited, it offers a novel perspective for uncovering the mechanisms underlying the onset and progression of ONFH.

The purpose of this study is to examine variations in GM composition and fecal metabolomic profiles in patients with SONFH and AONFH. These differences may provide insights into the mechanisms underlying ONFH induced by different etiologies. To our knowledge, this study represents the first analysis of GM and fecal metabolomic features in NONFH patients induced by steroid and alcohol.

## Materials and methods

### Study participants and sample collection

This study recruited a total of 68 individuals from October 2022 to April 2023 at the Chinese PLA General Hospital (Beijing, China). Among the 68 non-traumatic ONFH patients, 32 were classified as steroid-induced (12 males, 20 females) and 36 as alcohol-induced (21 males, 15 female) based on their medical history.

Inclusion criteria: (a) definite diagnosis of ONFH based on radiographic examinations and meeting the diagnostic criteria proposed by Mont et al. [15]; (b) history of steroid- or alcohol-induced ONFH; (c) willingness to provide informed consent and allow the use of human fecal samples for testing, as well as the ability to provide qualified samples according to the standard procedure.

Exclusion criteria: use of probiotics or antibiotics within 4 weeks prior to sample collection; gastrointestinal surgery (within < 1 month before sample collection); uncertain diagnosis of ONFH; inability to confirm ONFH as steroid-induced or alcohol-induced based on medical history; refusal to provide informed consent or permission for the use of human tissue samples by the researchers; inability to collect samples as required or samples not meeting the quality standards.

Participants adhered to a standard diet and maintained regular bowel habits. Patients gathered morning fecal samples following defecation during their hospital visit. These collected samples are then quickly transported on dry ice within 10 min. All sample collection procedures are carried out by specialized personnel. The fecal samples were then stored at -80 °C for further processing.

### Sample DNA extraction and 16 S sequencing

Microbial DNA was extracted from fecal samples using the MagPure Stool DNA KF Kit B (MAGEN, MD5115-02B) following the manufacturer’s recommendations. The extracted DNA was quantified using the Qubit® dsDNA BR Assay kit (Invitrogen, USA) and its quality was assessed by running samples on a 1% agarose gel with liquid chromatography. The V3-V4 region of the 16 S rRNA gene was amplified using the specific primers 341 F (5’-ACTCCTACGGGAGGCAGCAG-3’) and 806R (5’-GGACTACHVGGGTWTCTAAT-3’). Both forward and reverse primers contained Illumina adapters, pad sequences, and linker sequences. Polymerase chain reaction (PCR) enrichment was carried out in 50 µL reactions containing 30 ng template, fusion PCR primers, and PCR master mix. The PCR products were purified using DNA selection beads (BGI, LB00V60) and eluted using elution buffer. Library identification was performed using the Agilent 2100 Bioanalyzer (Agilent, USA). Verified libraries were sequenced on the Illumina MiSeq platform (Novogene, Shenzhen, China) following the Illumina standard protocol, generating paired-end reads of 2 × 300 bp. Subsequently, QIIME2 was employed for bioinformatics analysis.

### Sequencing data analysis

After Illumina MiSeq sequencing, raw data were obtained. Reads matching the primers were processed using the cutadapt software (v2.6) to trim the primers and adapter contaminants, resulting in fragments of the target region. Low-quality regions were removed using a sliding window approach: a window size of 30 bp was set, and if the average quality within the window was below 20, sequences were trimmed from the window start. Reads with a final length less than 75% of the original read length were removed. Reads containing ‘N’ were eliminated, and reads with low complexity (consecutive 10 ATCG) were also removed to obtain Clean data [16]. Subsequently, sequence assembly was performed using FLASH (V1.2.11) [17].

The software USEARCH (v7.0.1090) was utilized to cluster the assembled tags into operational taxonomic units (OTUs) based on 97% sequence similarity [18]. The usearch_global method was employed to align all tags back to the OTU representative sequences, generating an abundance table for each sample’s OTUs. Once the OTU representative sequences were obtained, the RDP classifier software (v2.2) was used to annotate them against a reference database for species annotation, with a confidence threshold set at 0.6. OTUs without annotation results or annotations not belonging to species within the scope of analysis were filtered out, leaving the remaining OTUs for subsequent analysis.

Within our samples, microbial α-diversity was computed using mothur software (v.1.31.2). The complexity of species diversity was evaluated by α-diversity indices such as Chao1 index. For microbial β-diversity analysis, QIIME software (v1.80) was utilized. β-diversity analysis suggests differences in species complexity between samples, using QIIME software (v1.80) to calculate unweighted UniFrac distances [19].Principal coordinate analysis (PCoA) visualized the distance matrix between all samples, conducted using the stats R package. ADONIS analyses were employed to evaluate significant differences in β-diversity among different groups.

The Linear Discriminant Analysis Effect Size (LEfSe) tool was employed for linear discriminant analysis. Metastats analysis using the stats R package was also conducted to determine *p*-values for distinguishing bacterial taxa with significant differences in abundance. Bacterial taxa with *p*-values < 0.05 and log linear discriminant analysis (LDA) score > 2 were considered significant. Only bacterial taxa meeting these criteria were displayed.

### Metabolomic analysis

In this study, a Waters UPLC I-Class Plus coupled with a Q Exactive high-resolution mass spectrometer (Thermo Fisher Scientific, USA) was used for the separation and detection of metabolites. The samples were thawed at 4 °C, 25 mg was taken and placed in a 1.5 mL Eppendorf tube by the researchers. Add 800 µL of extraction solution + 10 µL internal standard. Bead beating was carried out in a tissue grinder (50 Hz, 5 min), followed by sonication for 10 min in a -20 °C freezer for 1 h. Afterward, the samples were centrifuged at 25,000 g for 15 min at 4 °C. A volume of 600 µL of the supernatant was collected, freeze-dried using a cold vacuum concentrator, and reconstituted. The mixture was vortexed for 1 min, sonicated for 10 min in a 4 °C water bath, and centrifuged again (25,000 g, 15 min, 4 °C). The supernatant was then collected for analysis. To create a quality control (QC) sample for assessing the repeatability and stability of the LC-MS analysis process, 50 µL of supernatant from each sample was mixed.

A Waters UPLC I-Class Plus system coupled with a Q Exactive high-resolution mass spectrometer (Thermo Fisher Scientific, USA) was employed for non-targeted fecal metabolomic analysis. Chromatographic condition: A BEH C18 chromatographic column (1.7 μm, 2.1*100 mm, Waters, USA) was utilized.

### Data analysis

The initial data for mass spectrometry is entered into Compound Discoverer 3.3 software (Thermo Fisher Scientific, USA). After analyzing the mass spectrum data through online databases such as BMDB (BGI Metabolome Database), a data matrix containing details such as metabolite peak area and identification results is generated. Subsequently, metaX was employed for data pre-processing and subsequent analysis.

Data preprocessing involved the following procedures:

1. Normalization was performed using the PQN method to derive relative peak areas.
2. Batch effect correction was implemented using QC-RLSC.
3. Elimination of compounds with a CV of relative peak areas exceeding 30% in all QC samples.

Principal components analysis (PCA) and orthogonal partial least squares discriminant analysis (OPLS-DA) utilized logarithmic transformation and Pareto scaling. Apart from multivariate statistical approaches, univariate analysis employing Student’s *t*-test was employed to pinpoint metabolites exhibiting significant changes in NONFH patients.

In the OPLS-DA, metabolites with VIP values exceeding 1 and a significance level of *P* < 0.05 in the univariate analysis were deemed significantly altered. Additionally, metabolites with modified abundance were associated with their respective biochemical pathways through pathway enrichment and analysis grounded in the Human Metabolome Database (HMDB). KEGG metabolic pathways were employed for pathway functional annotation, facilitating the recognition of the principal biochemical metabolic pathways in which the metabolites participate.

### Statistical analysis

Spearman correlation analysis was employed to calculate the correlation coefficient (Corr) matrix and correlation *P* value matrix for the levels of fecal metabolites and the relative abundances of genera. The correlation was assessed specifically for genera and metabolites with *P* < 0.05. To mitigate type I errors and address multiple comparisons, a two-tailed *P* < 0.05 was pre-determined for statistical significance in all tests.

The unpaired Student’s *t*-test was employed for comparing parameter data between groups. Data analysis was conducted using the Statistics Package for Social Sciences (SPSS for Windows, version 27.0; SPSS Inc, Chicago, IL, USA). Continuous data are expressed as mean ± standard deviation (SD). Fisher’s exact test, Chi-square, or Chi-square test with Yates correction, as appropriate, were utilized to compare gender, smoker status, diabetes, hypertension, side of ONFH, and ARCO stage between the two groups.

## Results

### Participant characteristics

Table 1 shows the characteristics of the SONFH and AONFH patients. Except BMI, there were no statistically significant differences in terms of gender, age, smoking, side of osteonecrosis, ARCO stage, duration of osteonecrosis and comorbidities of hypertension and diabetes mellitus between two groups (*p* > 0.05).

**Table 1:** Demographic and clinical characteristics of SONFH patients and AONFH patients

Characteristics	SONFH	AONFH	*P*-value	F/X^2^
Number of patients	32	36		
Gender (male/female)	12/20	21/15	0.086	
Age (years)	45.91 ± 15.75	50.17 ± 12.68	0.074	3.29
BMI (kg/m2)	24.42 ± 4.08	25.91 ± 2.55	0.022	5.47
Smoker n (%)	13(40.63%)	22(61.11%)	0.092	
Diabetes	1	3	0.616	
Hypertension	4	4	1.000	
**Characteristics of ONFH**				
Number of hips	61	63		
Side (right: left)	31:30	31:32	0.857	0.03
Timing since the onset of the ONFH, months	53.25 ± 75.44	60.28 ± 65.46	0.854	0.03
Harris score				
VAS score				
**Steroid dosage**				
Steroid pulse therapy, n (%)	16(50.00%)	NA	NA	
Duration of Steroid administration, years	4.78 ± 6.54	NA	NA	
**Steroid treatment**				
Drug species	Prednisone	NA	NA	
	Methylprednisolone	NA	NA	
	Dexamethasone	NA	NA	
**Alcohol consumption**				
Duration of alcohol consumption (years)	NA	24.67 ± 11.47	NA	
Weekly ethanol consumption (g/week)	NA	2412.50 ± 1435.09	NA	
Ethanol drink-years ((g/week) × years)	NA	59020.83 ± 45448.25	NA	
**ARCO STAGE**				0.185
Non-osteonecrosis	3	9		
STAGE I	9	7		
STAGE II	7	6		
STAGE IIIA	7	5		
STAGE IIIB	13	25		
STAGE IV	25	20		

### Changes in GM in patients with AONFH and SONFH

**Fig. 1:**
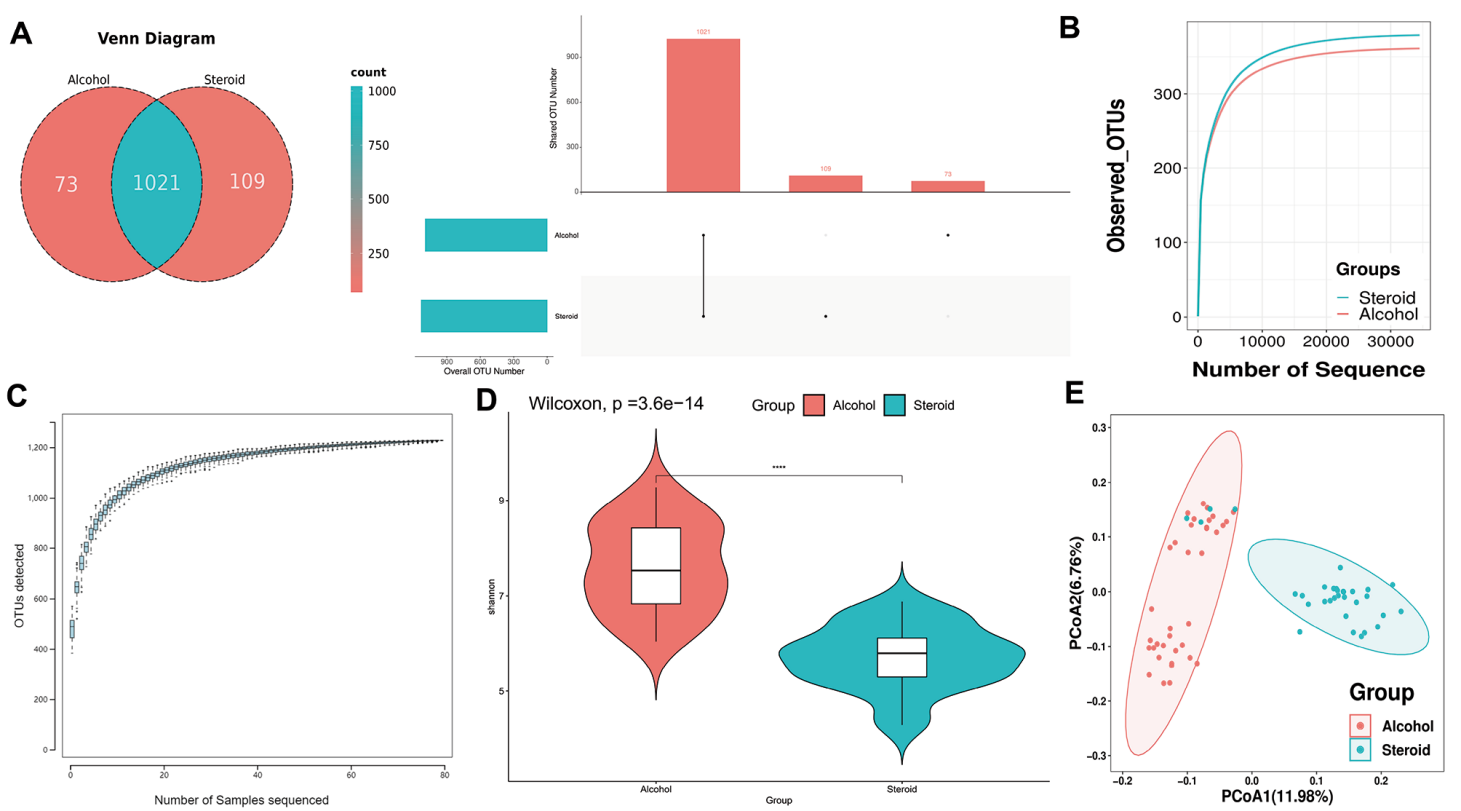
The difference in the composition of the GM in fecal samples between SONFH and AONFH patients. (**A**) Venn diagrams and an upset view illustrating the shared or unique distribution of OTUs in SONFH and AONFH patients. (**B**) By progressively increasing the sequencing depth of randomly selected samples, sparse curves were obtained for each group. As the sequencing depth of the samples advanced, the curves neared saturation, indicating that the sequencing data were sufficient and stable. (**C**) The cumulative species curve showed a trend of leveling off at the end, indicating an adequate sample size. (**D**) Alpha diversity differences between the two groups, as indicated by the Shannon index, revealed meaningful distinctions in alpha diversity between SONFH and AONFH (*P* < 0.05). (**E**) PCoA sorting based on Bray-Curtis distances from 16 S rDNA sequencing data showed significant separation between the two groups, suggesting notable variations in beta diversity between the two groups

A total of 68 NONFH patients were categorized based on the etiology into AONFH (36 cases) and SONFH (32 cases). From fecal samples of non-traumatic ONFH patients, a total of 3,201,069 valid tags were obtained, with an average of 47,075 per sample (ranging from 37,324 to 53,967). Sequences were clustered into operational taxonomic units (OTUs) at 97% identity, resulting in a total of 1203 OTUs. A Venn diagram was generated to assess the presence of shared and unique OTU patterns among the groups (Fig. 1A). The two groups have 1021 overlapping OTUs, with 109 OTUs exclusive to the SONFH and 73 OTUs exclusive to the AONFH. After obtaining the representative sequences of OTUs, species annotation was performed using the RDP classifier software against the Greengene V201305 database. The sequencing and samples were deemed sufficient for identifying taxonomic groups, as indicated by the rarefaction curve (Fig. 1B) and species accumulation curve (Fig. 1C).

The alpha diversity indices, including the Shannon index (Fig. 1D) and Simpson index (Supplementary Figure S1), indicate significant differences between the two groups (*P* < 0.05). The alpha diversity of the microbiota associated with SONFH is reduced. Compared to the steroid group, the alcohol group exhibits higher microbial richness indices, Chao1, and microbial coverage indices, Goods_coverage, although there is no statistical difference between the two groups (Supplementary Figure S1). Significant differences exist in the alpha diversity of microbial communities between the two groups. In beta diversity analysis, gut microbial communities were distinguishable between the two groups through PCoA (Fig. 1E), as confirmed by ADONIS analysis (*p* < 0.01) and ANOSIM analysis (*p* < 0.01) (Supplementary Table S1).

**Fig. 2:**
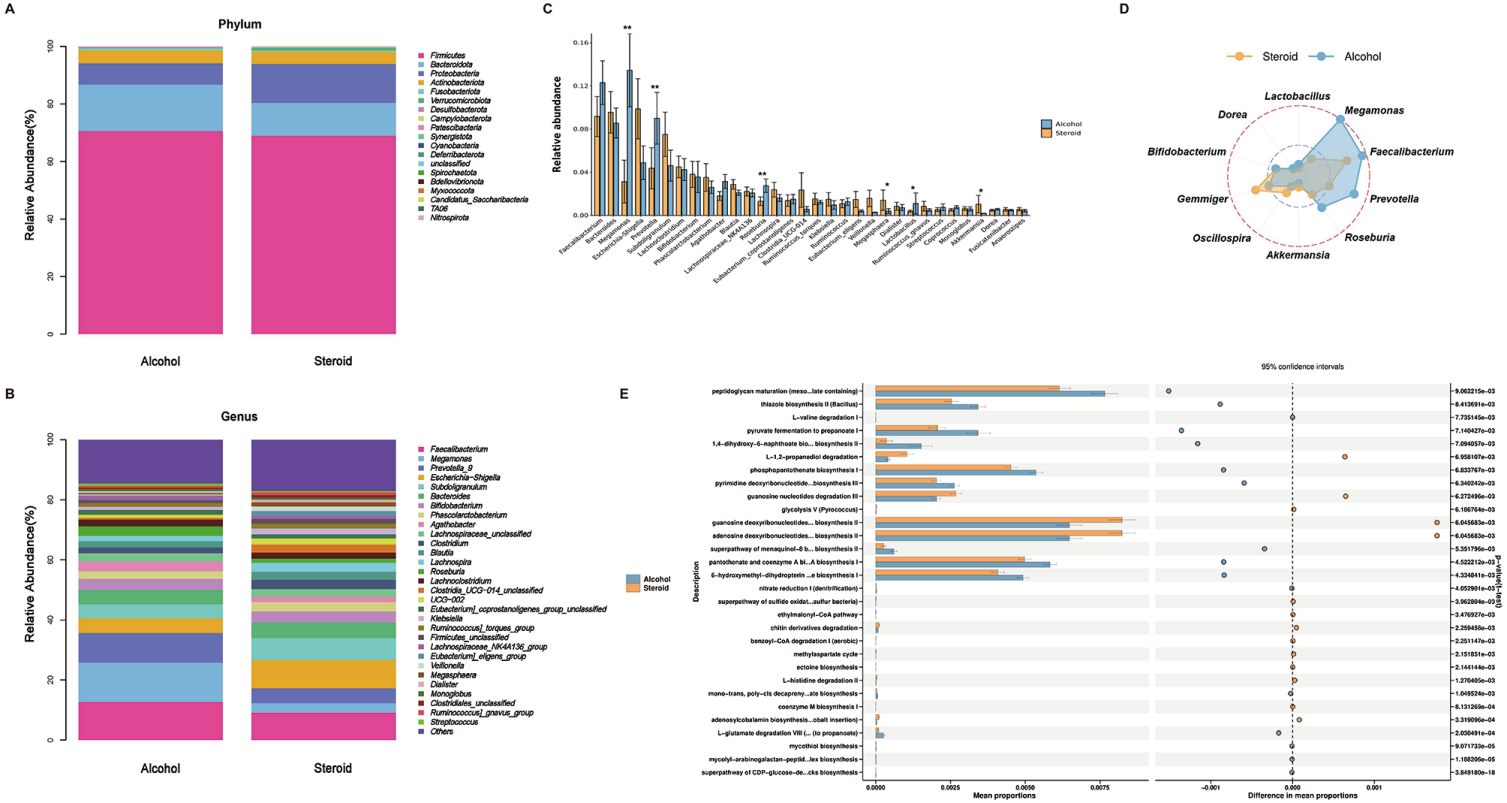
Classification distribution of fecal microbiota in SONFH and AONFH patients. (**A**) Stacked bar chart illustrating the phylum-level bacterial proportions and composition in both groups. (**B**) Stacked bar chart depicting the genus-level bacterial proportions and composition in both groups. (**C**) At the genus level, grouped bar chart displaying the differentially abundant microbiota between SONFH and AONFH. (**D**) Radar chart depicting the distinct taxonomic compositions of the six significantly different genera. The inner green circle signifies a relative abundance of 0.0014211, the middle blue circle represents a relative abundance of 0.05, and the outer red circle represents a relative abundance of 0.12973.*Gemmiger* and *Akkermansia* are dominant in the steroid group, while *Megamonas*, *Prevotella*, and others are more abundant in the alcohol group. (**E**) Bar chart depicting the KEGG pathways that exhibit significant differences between SONFH (yellow) and AONFH (blue)(*P*<0.05)

Then, the composition and taxonomic structure of fecal microbiota were evaluated at different taxonomic levels. A total of 19 phyla, 37 classes, 79 orders, 154 families, and 422 genera were annotated. Figure 2A-B display the top 30 most abundant taxonomic units identified in each group at the phylum and genus levels. *Firmicutes* dominated the GM, followed by *Bacteroidota*. At the genus level, *Faecalibacterium*, *Megamonas*, *Prevotella*, and *Escherichia − Shigella* were the predominant taxa. *Clostridia*, *Negativicutes*, *Bacteroidia*, *Gammaproteobacteria*, and *Actinobacteria* were major classes; *Oscillospirales*, *Lachnospirales*, and *Bacteroidales* were major orders; *Lachnospiraceae*, *Ruminococcaceae*, *Enterobacteriaceae*, *Selenomonadaceae*, and *Prevotellaceae* were major families (Supplementary Figure S2).

We utilized the Mann-Whitney U test to analyze inter-group differences in microbial communities between the two groups (*p* < 0.05), revealing significant differences in abundance for six functionally important bacterial genera. Among them, *Megamonas*, *Prevotella*, *Roseburia*, and *Lactobacillus* exhibited significantly higher abundance in the alcohol group, while *Gemmiger* and *Akkermansia* showed relatively higher abundance in the steroid group (Fig. 2C). At the genus level, *Megamonas* dominated in the alcohol group, while *Gemmiger* was predominant in the steroid group (Fig. 2D).

We utilized PICRUSt to analyze the metabolic and functional pathway potentials in the gut microbiome of SONFH and AONFH patients. A comparison of KEGG pathways between the steroid and alcohol groups revealed significant differences, with 166 KEGG categories showing distinct abundances (Fig. 2E). In patients with SONFH, KEGG pathways, including Coenzyme M Biosynthesis, Glycolysis, Guanosine Nucleotides Degradation, L-histidine Degradation, and L-valine Degradation, were significantly elevated. In patients with AONFH, KEGG pathways were mainly concentrated in L-glutamate Degradation VIII (to propanoate), Pantothenate and Coenzyme A Biosynthesis, Peptidoglycan Maturation, Phosphopantothenate Biosynthesis, and Pyruvate Fermentation, among others.

**Fig. 3:**
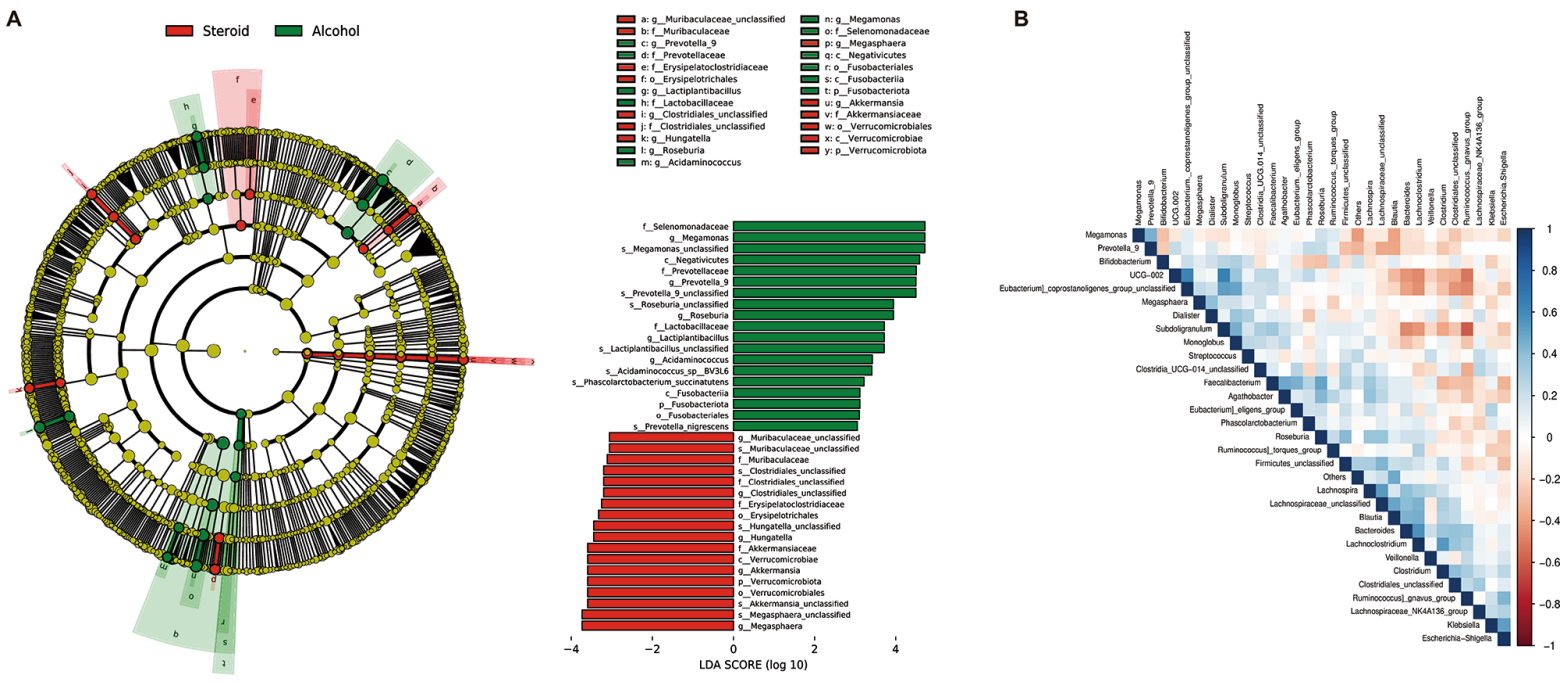
Taxonomic differences between SONFH and AONFH patients. (**A**) The LEfSe branching diagram and LDA score revealed differences in the composition of taxa, with bacterial taxa notably enriched in the alcohol group (green) and the steroid group (red). (**B**) This heatmap describes the correlations between the compositions of different taxa. Red denotes an inverse correlation, whereas blue signifies a positive correlation

To identify differences in abundance among non-traumatic ONFH patients, we compared the AONFH group and the SONFH group, followed by conducting LEfSe analysis. Using LEfSe, our discriminant analysis revealed numerous key taxonomic groups that significantly differed between the AONFH and SONFH groups (LDA score > 2, *p* < 0.05). The findings indicated an enrichment of 19 bacterial taxa in AONFH patients, while 18 bacterial taxa were enriched in SONFH patients. The LEfSe analysis generated a hierarchical branching diagram at 6 different taxonomic levels (from domain to genus). *Verrucomicrobiota* plays a crucial role in the GM of SONFH patients. Additionally, *Akkermansia*, *Megasphaera*, *Hungatella*, *Clostridiales*, and *Muribaculaceae* also significantly influence the GM of SONFH patients. In the GM of AONFH patients, *Fusobacteriota* plays a key role. Moreover, *Megamonas*, *Acidaminococcus*, *Roseburia*, *Lactiplantibacillus*, and *Prevotella* also have a significant impact on AONFH patients (Fig. 3A). Figure 3B shows a heatmap of bacterial correlations in the steroid and alcohol groups.

### Metabolic changes in patients with AONFH compared to SONFH

**Fig. 4:**
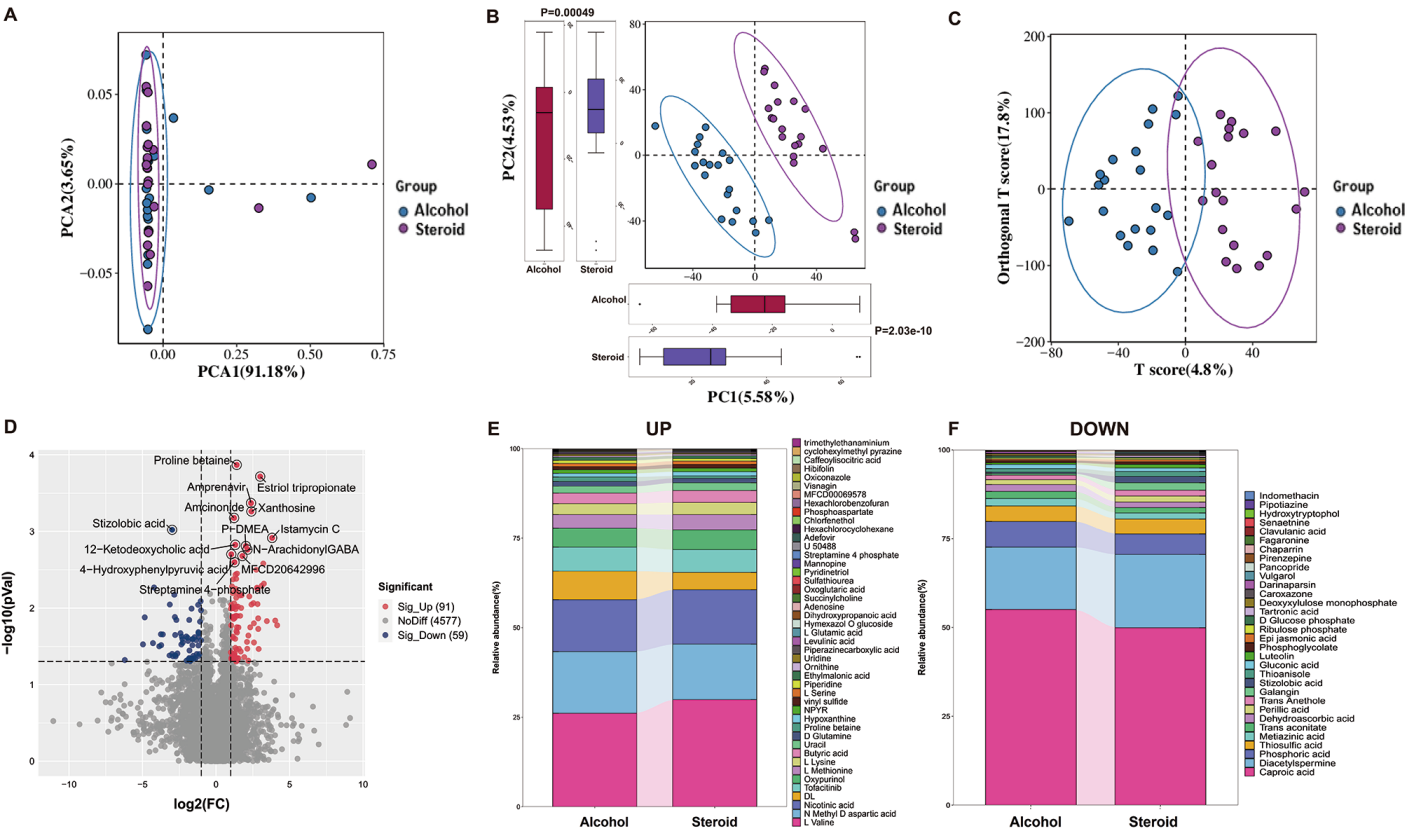
Overall patterns of fecal metabolomes in SONFH and AONFH patients. (**A**) Principal component analysis (PCA) plot based on non-targeted LC-MS/MS data of fecal samples from the steroid and alcohol groups, depicting the metabolic spectra and features. (**B**) Scatter plot of PLS-DA identifying metabolic distinctions and separations between the steroid and alcohol groups. *p*-values of 2.03e − 10 and 0.00049 were obtained for components 1 and 2, respectively. (**C**) Scatter plot of OPLS-DA showing metabolic distinctions and separations between two groups. (**D**) Volcano plot illustrating significant changes in fecal metabolites. Metabolites with a *p*-value <0.05 were deemed significantly different. Upward points suggest a significant rise in the abundance of fecal metabolites in SONFH compared to AONFH; downward points indicate a significant decrease in the abundance of metabolites in SONFH; Some metabolites are labeled with corresponding annotations. (**E**) Percentage stacked bar chart showing the relative abundance and percentage of significantly increased metabolites in the steroid group compared to both groups. (**F**) Percentage stacked bar chart showing the proportionate abundance and percentage of significantly decreased metabolites in SONFH compared to AONFH

Current metabolomic studies on ONFH are limited, mainly focusing on urine, plasma, and bone tissue samples [20, 21]. However, there has been minimal research on fecal samples from ONFH patients. Therefore, we conducted a metabolomic analysis on fecal samples to explore metabolic alterations in individuals with non-traumatic ONFH. Based on fecal metabolomic data, PCA, partial least squares discriminant analysis (PLS-DA), and OPLS-DA cluster analyses were employed to assess the differentiation between the steroid and alcohol groups. As evident in the PCA plot, there is an overlap of samples from different groups, and SONFH patients cannot be distinctly separated from AONFH patients (Fig. 4A). Nevertheless, based on the fecal metabolome, the PLS-DA scatter plot demonstrates a clear separation between the steroid and alcohol groups. Significant differences in scores between SONFH and AONFH patients are evident in components 1 and 2 (Fig. 4B). Conversely, individuals in the steroid group exhibit differences from those in the alcohol group, as indicated in the OPLS-DA score plot (Fig. 4C). The validity of this model is confirmed by the evident separation after randomization (*n* = 200) (Supplementary Figure S3). By comparing the metabolites between the two groups (AONFH group and SONFH group) using the criteria of *p* < 0.05, VIP > 1, and fold change (FC ≥ 1.2 or ≤ 0.83), 150 metabolites were found to have significant differences between the groups, with 59 metabolites significantly upregulated and 91 metabolites significantly downregulated (Fig. 4D). For detailed metabolite composition, refer to Fig. 4E-F for significantly upregulated and downregulated metabolites.

For a more precise characterization of the metabolic phenotypes in SONFH and AONFH, we conducted a comparison of metabolites between the two patient groups. Fecal samples from AONFH patients showed elevated levels of L-Lysine, Oxoglutaric acid, L-Serine, L-Glutamic acid, L-Valine, L-(-)-Methionine, Phosphoaspartate, (2R)-2,3-Dihydroxypropanoic acid, Phenylpyruvic acid, Xanthosine, and 4-Hydroxyphenylpyruvic acid. In contrast, fecal samples from SONFH patients exhibited increased levels of Gluconic acid, Phosphoric acid, ribulose 5-phosphate, and phosphoglycolate (Table 2).

**Table 2:** Identified differential Fecal metabolites between SONFH patients and AONFH patients

Metabolites	Mean SONFH	Mean AONFH	VIP	*P*-value^a^	FC
L-Lysine	1.11E + 08	1.63E + 08	1.73	0.047	1.47
Oxoglutaric acid	1.29E + 06	3.30E + 06	2.33	0.010	2.57
L-Serine	2.97E + 07	4.77E + 07	1.73	0.021	1.61
L-Glutamic acid	5.50E + 06	9.14E + 06	1.89	0.017	1.66
L-Valine	9.72E + 08	1.40E + 09	2.72	0.034	1.44
L-(-)-Methionine	1.44E + 08	2.00E + 08	1.41	0.017	1.39
D-Glutamine	4.06E + 07	7.08E + 07	2.25	0.014	1.75
Phosphoaspartate	2.70E + 05	1.24E + 06	1.83	0.049	4.59
(2R)-2,3-Dihydroxypropanoic acid	2.88E + 06	7.18E + 06	2.69	0.004	2.50
Phenylpyruvic acid	1.95E + 06	4.28E + 06	2.00	0.006	2.19
Xanthosine	3.18E + 05	1.68E + 06	3.55	0.001	5.29
4-Hydroxyphenylpyruvic acid	1.01E + 06	2.07E + 06	1.66	0.002	2.05
Gluconic acid	1.50E + 06	8.95E + 05	1.21	0.042	0.60
Phosphoric acid	9.49E + 06	4.21E + 06	2.84	0.024	0.44
ribulose 5-phosphate	6.31E + 05	2.55E + 05	1.35	0.040	0.40
phosphoglycolate	1.05E + 06	3.94E + 05	1.92	0.046	0.38

VIP, variable importance in the projection; FC, fold change.^a^*P*-value was calculated by Student’s *t*-test

### Correlation of the GM and metabolic phenotype in patients with NONFH

**Fig. 5:**
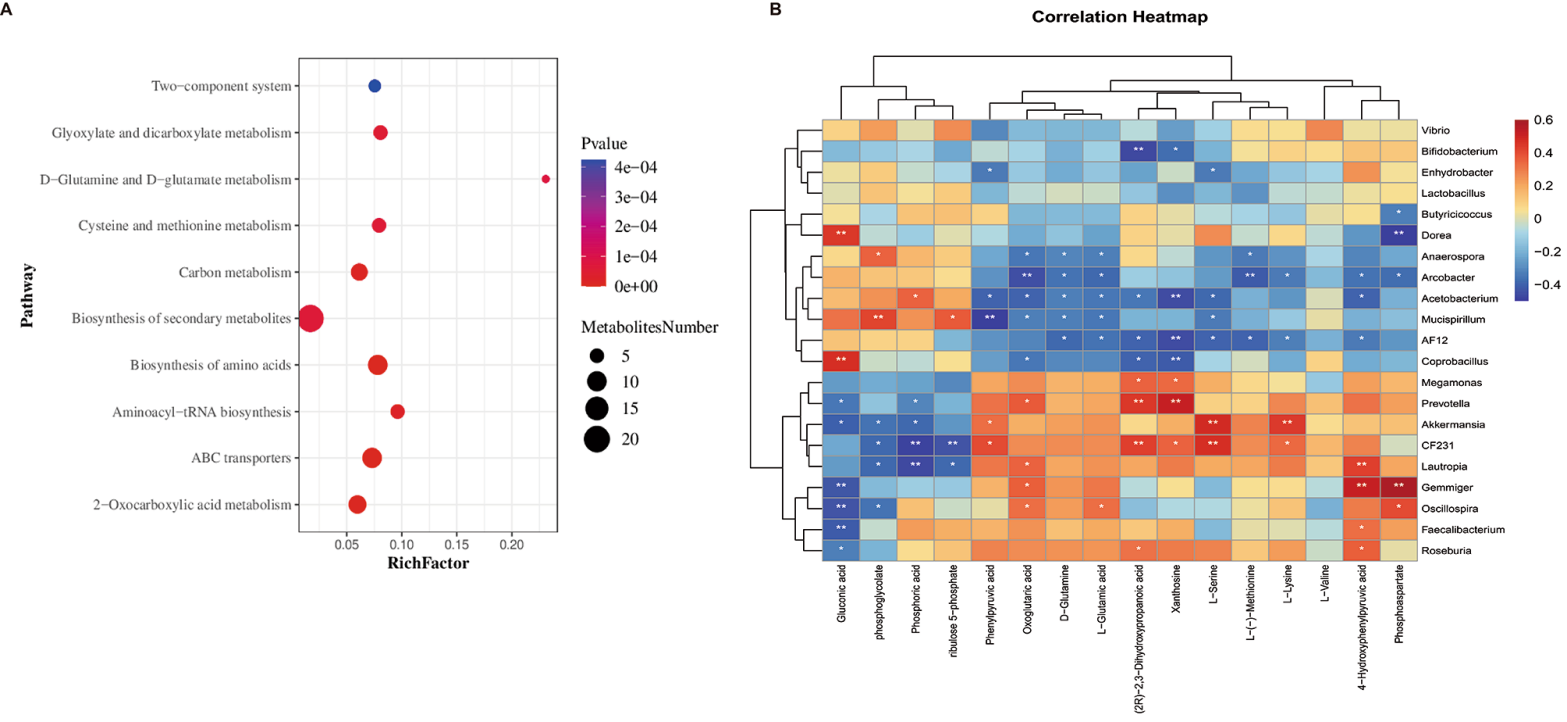
Joint analysis of fecal metabolome and microbiome in SONFH and AONFH patients. (**A**) Bubble chart illustrating differential metabolic pathways between SONFH and AONFH patients. (**B**) Relationship between GM and fecal metabolites in patients with SONFH and AONFH. Red denotes a positive correlation between microbial taxa and metabolites, while blue indicates a negative correlation. * *p* < 0.05; ** *p* < 0.01, Spearman correlation

To determine the metabolic pathways involved in SONFH and AONFH, we conducted KEGG annotation. Comparative analysis of the two groups identified 10 pathways with differential metabolite abundance. The results of the pathway analysis were displayed as a bubble plot (Fig. 5A), revealing pathways such as Biosynthesis of amino acids, ABC transporters, 2-Oxocarboxylic acid metabolism, and Carbon metabolism. Pathways including 2-Oxocarboxylic acid metabolism, Aminoacyl-tRNA biosynthesis, Cysteine and methionine metabolism, and D-Glutamine and D-glutamate metabolism showed varying metabolite abundance between SONFH and AONFH patients.

To investigate whether the composition of GM is correlated with the fecal metabolic phenotype in patients with non-traumatic ONFH, Spearman correlation analysis was conducted between the steroid group and the alcohol group (Fig. 5B). The results revealed the following correlations: *Akkermansia* was positively correlated with L-Lysine (*P* = 0.0054, *r* = 0.440), L-Serine (*P* = 0.0019, *r* = 0.487), and Phenylpyruvic acid (*P* = 0.042, *r* = 0.327), and negatively correlated with Gluconic acid (*P* = 0.010, *r*=-0.408), Phosphoric acid (*P* = 0.016, *r*=-0.384), and phosphoglycolate (*P* = 0.027, *r*=-0.356). *Megamonas* showed a positive correlation with Xanthosine (*P* = 0.034, *r* = 0.342) and (2R)-2,3-Dihydroxypropanoic acid (*P* = 0.030, *r* = 0.349). *Roseburia* exhibited positive correlations with (2R)-2,3-Dihydroxypropanoic acid (*P* = 0.048, *r* = 0.319) and 4-Hydroxyphenylpyruvic acid (*P* = 0.026, *r* = 0.358), but a negative correlation with Gluconic acid (*P* = 0.046, *r*=-0.322). *Prevotella* showed a positive correlation with Oxoglutaric acid (*P* = 0.021, *r* = 0.369), Xanthosine (*P* = 0.001, *r* = 0.512), and (2R)-2,3-Dihydroxypropanoic acid (*P* = 0.004, *r* = 0.452), but had a negative correlation with Gluconic acid (*P* = 0.027, *r*=-0.352) and Phosphoric acid (*P* = 0.046, *r*=-0.322).

Further correlation analysis between GM and fecal metabolites was conducted based on the grouping of SONFH and AONFH, revealing significant differences (Supplementary Figure S4). Particularly noteworthy is the significant correlation in the alcohol group, where *Lactobacillus* showed a significant correlation with phosphoglycolate, D-Glutamine, L-Glutamic acid, L-Serine, L-Lysine, and ribulose 5-phosphate (*P* < 0.05), whereas in the steroid group, *Lactobacillus* showed no significant correlation with these metabolites (*P* > 0.05). Interestingly, Oxoglutaric acid exhibited a significant correlation with *Lactobacillus* in the steroid group (R^2^_(S)_ = 0.094, *P* = 0), while there was no significant correlation in the alcohol group (R^2^_(A)_ = 0.012, *P* = 1).

## Discussion

In this study, we observed significant differences in the composition of the intestinal microbiota between patients with SONFH and AONFH. Notably, the relative abundance of *Akkermansia* differed in the fecal samples of the two groups, being significantly depleted in the feces of AONFH patients. Although the relationship between *Akkermansia* and steroid use remains unproven, it has been closely associated with alcohol consumption [22]. Previous studies have shown a significant reduction in *A. muciniphila* levels in the feces of alcohol abusers compared to healthy individuals [23], consistent with our findings.Interestingly, in mice fed with ethanol, oral supplementation of *A. muciniphila* could restore its depletion [24]. Furthermore, oral supplementation of *A. muciniphila* has been shown to significantly reduce total cholesterol levels in plasma and reverse metabolic disruptions induced by a high-fat diet [25]. Abnormal lipid metabolism is considered a potential factor underlying non-traumatic ONFH [26]. Yan et al. used plasma lipidomic analysis to investigate changes in lipid profiles among ONFH patients with different etiologies [27]. Their results indicated that patients with AONFH exhibited the highest levels of lipid species, particularly triacylglycerol compounds, suggesting a potential link with the depletion of *Akkermansia* observed in our study.

The significant reduction of *Lactobacillus spp.* in patients with SONFH is consistent with the findings of Chen et al. [28]. Their study confirmed the association between the loss of *Lactobacillus* and its extracellular vesicles (EVs) with the development of SONFH. *Lactobacillus* has been shown to mitigate SONFH by enhancing angiogenesis, promoting osteogenesis, and reducing cell apoptosis. Functional proteins within the EVs of *Lactobacillus* can exert pro-angiogenic, pro-osteogenic, and anti-apoptotic effects in the femoral head. However, exposure to steroids significantly decreases the abundance of *Lactobacillus*.

Previous studies have suggested that alcohol use can also reduce the abundance of *Lactobacillus spp.* [29, 30]. However, our results show a relative enrichment of *Lactobacillus* in the fecal samples of AONFH patients. This relative enrichment might be in contrast to the reduction caused by steroid exposure on *Lactobacillus spp.* On the other hand, Dubinkina et al. observed the expansion of *Bifidobacterium* and *Lactobacillus* in patients with alcoholic liver disease [31]. While our study did not include patients with alcoholic liver disease, it’s worth noting that ONFH can also be considered a condition related to alcohol abuse. In this pathological state, the elevation of *Lactobacillus spp.* might indeed be plausible.

It’s noteworthy that from a clinical perspective, the duration of steroid use in patients with SONFH (4.78 ± 6.54 years) was much shorter than the duration of alcohol consumption in AONFH patients (24.67 ± 11.47 years) (Table 1). This may suggest that AONFH often requires prolonged alcohol abuse to manifest.

Butyrate, produced by *Faecalibacterium spp.* and *Roseburia spp.*, has been demonstrated to regulate bone synthesis metabolism through CD8 + T cell-mediated Wnt10b, mediated by Treg cells [32]. The relative abundance of *Faecalibacterium* and *Roseburia* in the fecal samples of AONFH patients was significantly higher than in SONFH patients. This difference might be attributed, in part, to the distinct pathogenic durations between the two groups. Butyrate plays a crucial role in improving bone quality [33]. Osteoporosis (OP) and ONFH exhibit similar clinical and pathophysiological characteristics, and prior research has confirmed the correlation between non-traumatic ONFH and low bone mass [34].

Furthermore, there are differences in the genus *Prevotella* in the GM between SONFH and AONFH patients. The genus *Prevotella* is recognized as one of the three representative bacteria in the human GM and is also considered a core genus of the human gut microbiome [35, 36]. Studies by Zirk et al. and Hallmer et al. have shown high levels of *Prevotella* in medication-related jawbone necrosis lesions [37, 38]. Previous research has reliably linked *Prevotella* to inflammatory conditions. *Prevotella* predominantly stimulates Toll-like receptor 2, leading to the generation of Th17 inflammatory cytokines [39, 40]. Zou et al. found elevated levels of Th17 and IL-17 in osteonecrosis, which were positively correlated with the severity of pain [41].

*Megamonas* is a core genus in the GM and may be characteristic of Asian populations. It has been closely associated with ankylosing spondylitis (AS) and obesity [42]. Previous research has shown that *Megamonas* is one of the genera with the largest increase in relative abundance in patients with ankylosing spondylitis [43]. Additionally, *Prevotella* and *Megamonas* are significantly increased in obese patients, while weight loss or malnutrition is associated with decreased *Megamonas* abundance. The abundance of *Megamonas* is negatively correlated with the rate of weight loss [44, 45]. These findings appear to align with our clinical results, as the BMI of AONFH patients (25.91 ± 2.55) was significantly higher than that of SONFH patients (24.42 ± 4.08) (*P* = 0.022) (Table 1).

The above findings underscore the potential connections between intestinal microbiota and ONFH caused by different underlying factors. However, the changes in GM under conditions of alcohol or steroid-induced pathogenesis and their effects on the host’s metabolic phenotype are still unknown. This could be a focal point for explaining the mechanisms underlying ONFH caused by different factors. Therefore, we aimed to evaluate how the GM influences the fecal metabolic phenotype of patients with AONFH and SONFH. We observed altered levels of fecal metabolites in both groups of patients.

Oxidative stress refers to a disturbance in the cellular redox state and is considered one of the potential mechanisms of ONFH [46]. Glutathione (GSH) is a major cellular antioxidant that protects cells from oxidative stress [47]. In the results of our study, L-Lysine, L-Glutamic acid, and D-Glutamine were significantly elevated in AONFH. L-Lysine itself is a natural antioxidant that promotes the synthesis of glutathione, thus enhancing the body’s antioxidant capacity. Additionally, L-Lysine can regulate the activity of pyruvate dehydrogenase and, in turn, modulate the activity of intracellular peroxidases, helping to counteract oxidative damage caused by free radicals [48]. L-Lysine also contributes to bone metabolism and growth, stimulating the proliferation of osteoblasts, the main bone-forming cells [49, 50].

L-Glutamic acid and D-Glutamine are important substances in the synthesis of GSH [51, 52], which may suggest that oxidative stress might not play a significant role in the pathogenesis of AONFH. On the contrary, it could be an important mechanism in SONFH. We noticed that the enriched metabolites in the feces of AONFH patients mostly belong to the amino acid category. This might be related to steroid promoting protein degradation and amino acid utilization. Zhu et al. analyzed bone trabeculae in osteonecrotic femoral heads and found that amino acids were more profoundly affected than lipids, nucleotides, or pyrrolidines in these local tissues [53]. D-Glutamine and D-glutamate metabolism, as well as Cysteine and methionine metabolism, could be pathways associated with these findings.

Disturbed Energy Metabolism is another hypothetical mechanism proposed for the onset of ONFH [12]. In our findings, Gluconic acid, Phosphoric acid, ribulose 5-phosphate, and phosphoglycolate were decreased in AONFH patients, and these metabolites are closely related to Carbon metabolism. Energy metabolism sustains the fundamental activities of living organisms. It typically includes glycolysis (EMP), the tricarboxylic acid cycle (TCA), and the pentose phosphate pathway (PPP), as well as the breakdown of substances like amino acids and lipids to generate precursors for entry into the TCA cycle and other metabolic processes [54].

Gluconic acid is formed through glucose oxidation and exhibits a robust positive correlation with the abundance of *Coprobacillus* (*P* = 0.002). The pentose phosphate pathway (PPP) plays a crucial role in cellular metabolism, ensuring carbon homeostasis and supplying precursors for the biosynthesis of nucleotides and amino acids [55]. Ribulose 5-phosphate is utilized for nucleotide synthesis and the generation of glycolytic intermediates for amino acid synthesis. It is produced from fructose 6-phosphate, glyceraldehyde 3-phosphate, and sedoheptulose 7-phosphate, all of which are intermediates in the non-oxidative branch of the PPP [56]. Phosphoglycolate plays an important role in the C-2 cycle [57]. These results suggest that energy metabolism may play a relatively important role in the pathogenesis of AONFH.

Additionally, Oxoglutaric acid serves as a precursor to hydroxyproline-the most abundant amino acid in collagen proteins, and it plays a crucial role in both energy metabolism and amino acid metabolism [58]. It has protective effects on skeletal development at different stages of organism growth [59]. Prior research has demonstrated that Oxoglutaric acid can induce partial repair of bone and cartilage damage caused by excessive steroid use [60].

In contrast to previous studies, we distinguished NONFH into steroid and alcohol-induced categories for separate analysis. The significant differences in intestinal microbiota composition and fecal metabolic profiles between these two groups indicate the potential existence of distinct pathogenic mechanisms, warranting further in-depth investigation.

Despite our findings, limitations still exist in our study. Firstly, our study used human fecal specimens, and compared to animals, human specimens are subject to interference, which may have some unavoidable impacts. For instance, there were differences in the BMI of patients included in the steroid group compared to the alcohol group (*P* = 0.022). The primary reason for this difference could be due to the steroid group having a higher inclusion of females, whereas the alcohol group had more males. Although there was no statistical difference in gender composition between the two groups, the BMI of males is generally higher than that of females. Additionally, the abuse of alcohol further increased the BMI of patients in the alcohol group [61], which might be the reason for the differences observed. Moreover, the relationship between BMI and the gut microbiota and its metabolites is complex, requiring further research to ascertain. Currently, most studies have described the impact of the gut microbiota and its metabolites on BMI [62–64], but whether differences in BMI will affect the gut microbiota and its metabolites remains to be further studied.

Therefore, we strictly applied inclusion criteria, ensuring that only individuals affected by alcohol or steroid as a singular influencing factor were included. Additionally, the results obtained from human specimens are considered reliable compared to animal specimens. Secondly, due to the rigorous control of inclusion criteria, our sample size was relatively small, which is also a limitation. Lastly, the occurrence and development of osteonecrosis of the femoral head is a dynamic process. The samples we obtained only represent the patients’ status at a specific time point, which may not comprehensively reflect the influence of intestinal microbiota and its metabolites on osteonecrosis of the femoral head. However, according to the ARCO classification system, the majority of ONFH patients included in our study were classified as stage III and IV. Most of the included patients were preparing for joint replacement surgery and had a long history of steroid or alcohol use. The microbial and metabolic characteristics of the intestinal tract at this stage should be considered stable.

## Conclusion

Our findings, derived from multi-omics technology, suggested that steroids and alcohol exert differential effects on the GM and metabolites in patients. This divergence also manifests as the two inducers emphasizing different mechanisms to influence the progression of non-traumatic femoral head necrosis. Our findings offer a novel insight, aiming to alleviate the progression of ONFH and improve its non-surgical treatment outcomes by addressing microbial dysbiosis. This can be achieved through the improvement of dietary habits and lifestyle or supplementation with probiotics and prebiotics to restore the homeostasis of GM.

### Electronic supplementary material

Below is the link to the electronic supplementary material.

Supplementary Material 1

Supplementary Material 2

Supplementary Material 3

Supplementary Material 4

Supplementary Material 5

## Data Availability

Sequence data that support the findings of this study have been deposited in the MetaboLights with the primary accession code MTBLS5308 and NCBI with the primary accession code PRJNA1063217.

## Abbreviations

ONFH: Osteonecrosis of the femoral head
NONFH: Non-traumatic osteonecrosis of the femoral head
GM: Gut microbiota
THA: Total hip arthroplasty
LC-MS/MS: Liquid chromatography with tandem mass spectrometry
KEGG: Kyoto Encyclopedia of Genes and Genomes
SONFH: Steroid-induced osteonecrosis of the femoral head
AONFH: Alcohol-induced osteonecrosis of the femoral head
PCR: Polymerase Chain Reaction
OTUs: Operational taxonomic units
PCoA: Principal coordinate analysis
LEfSe: The linear discriminant analysis effect size
LDA: Linear discriminant analysis
QC: Quality control
PCA: Principal components analysis
PLS-DA: Partial least squares discriminant analysis
OPLS-DA: Orthogonal partial least squares discriminant analysis
HMDB: Human Metabolome Database
VIP: Variable importance in the projection
FC: Fold change
EVs: Extracellular vesicles
OP: Osteoporosis
AS: Ankylosing spondylitis
PPP: Pentose phosphate pathway
TCA: Tricarboxylic acid cycle
GSH: Glutathione
