# Supplementary figures and images for: Non-traumatic osteonecrosis of the femoral head induced by steroid and alcohol exposure is associated with intestinal flora alterations and metabolomic profiles
Source: J Orthop Surg Res. 2024 Apr 12;19:236. doi: 10.1186/s13018-024-04713-z (PMC11015587; doi:10.1186/s13018-024-04713-z)

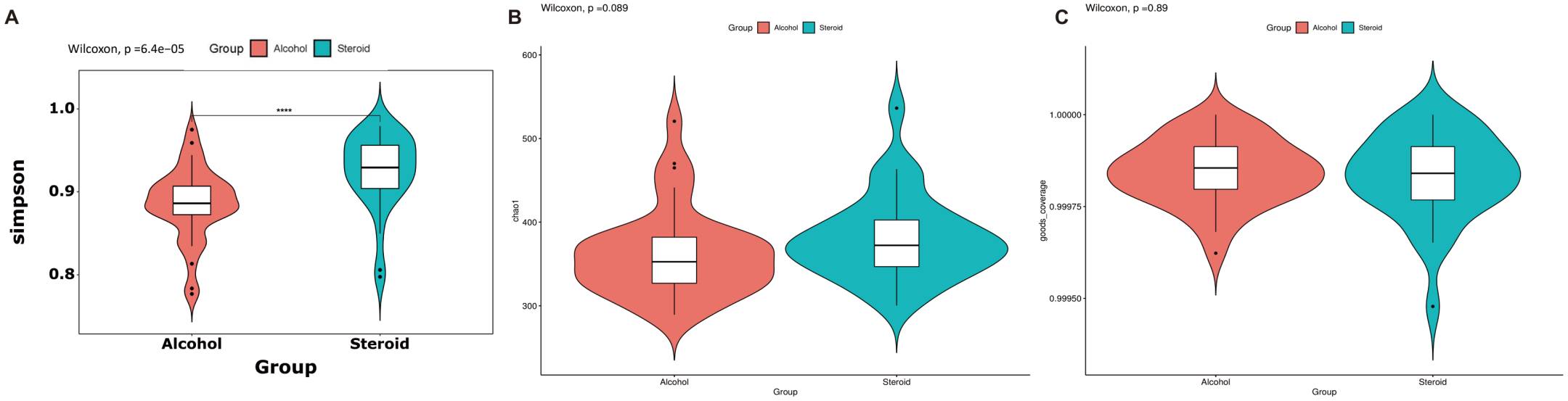

Supplement: Supplementary file 2 — Supplementary Material 2 [file 13018_2024_4713_MOESM2_ESM.jpg]

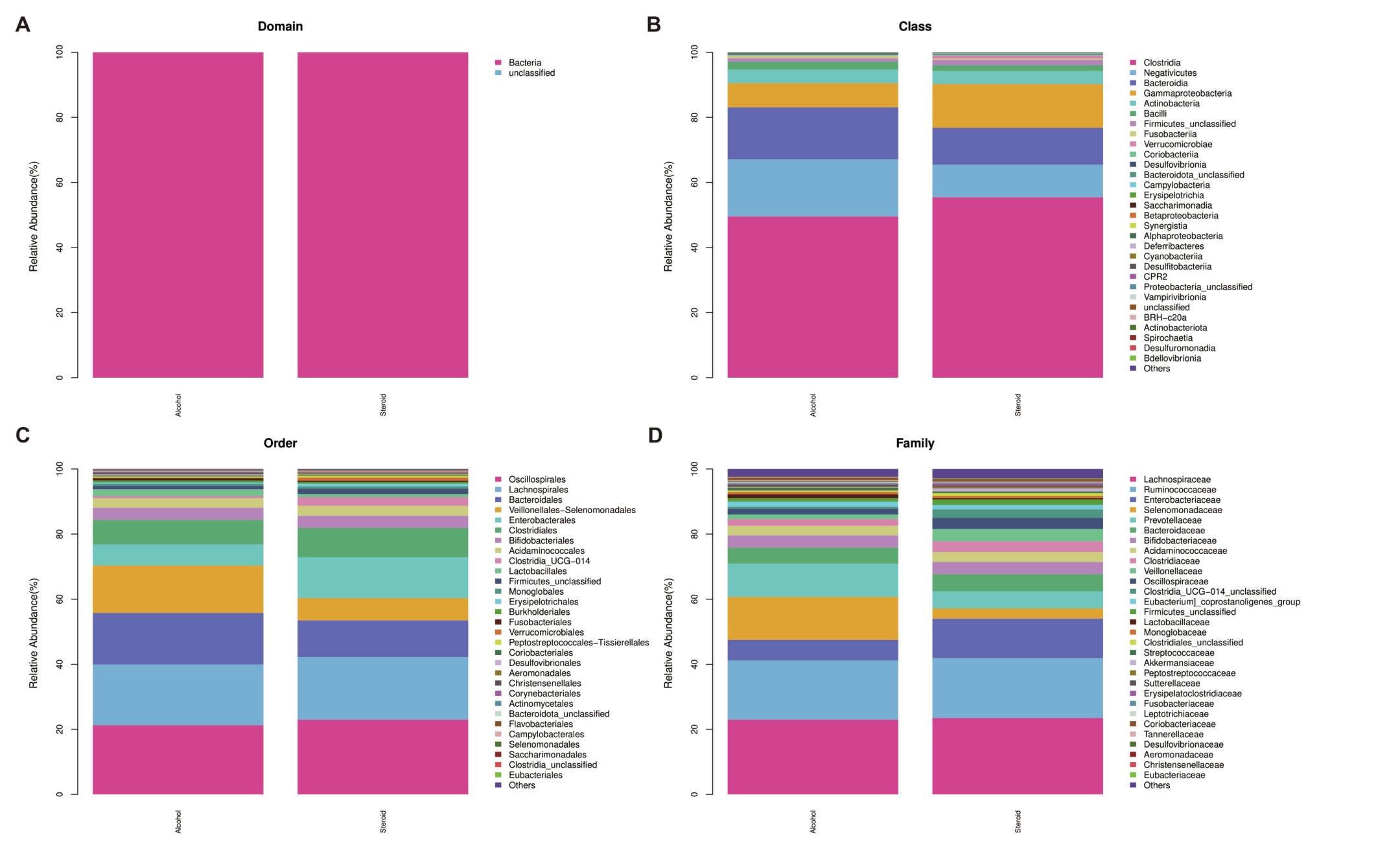

Supplement: Supplementary file 3 — Supplementary Material 3 [file 13018_2024_4713_MOESM3_ESM.jpg]

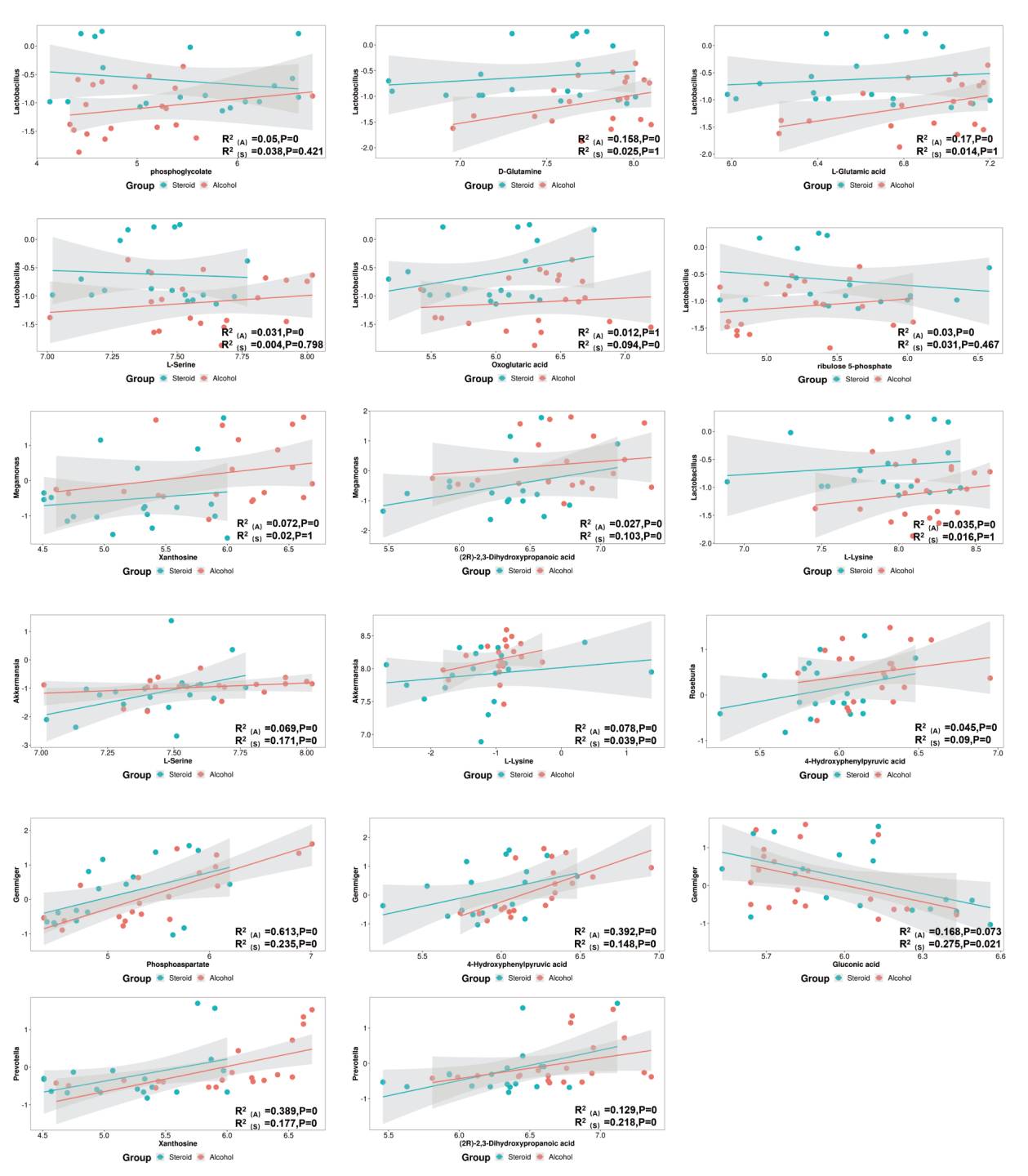

Supplement: Supplementary file 4 — Supplementary Material 4 [file 13018_2024_4713_MOESM4_ESM.jpg]

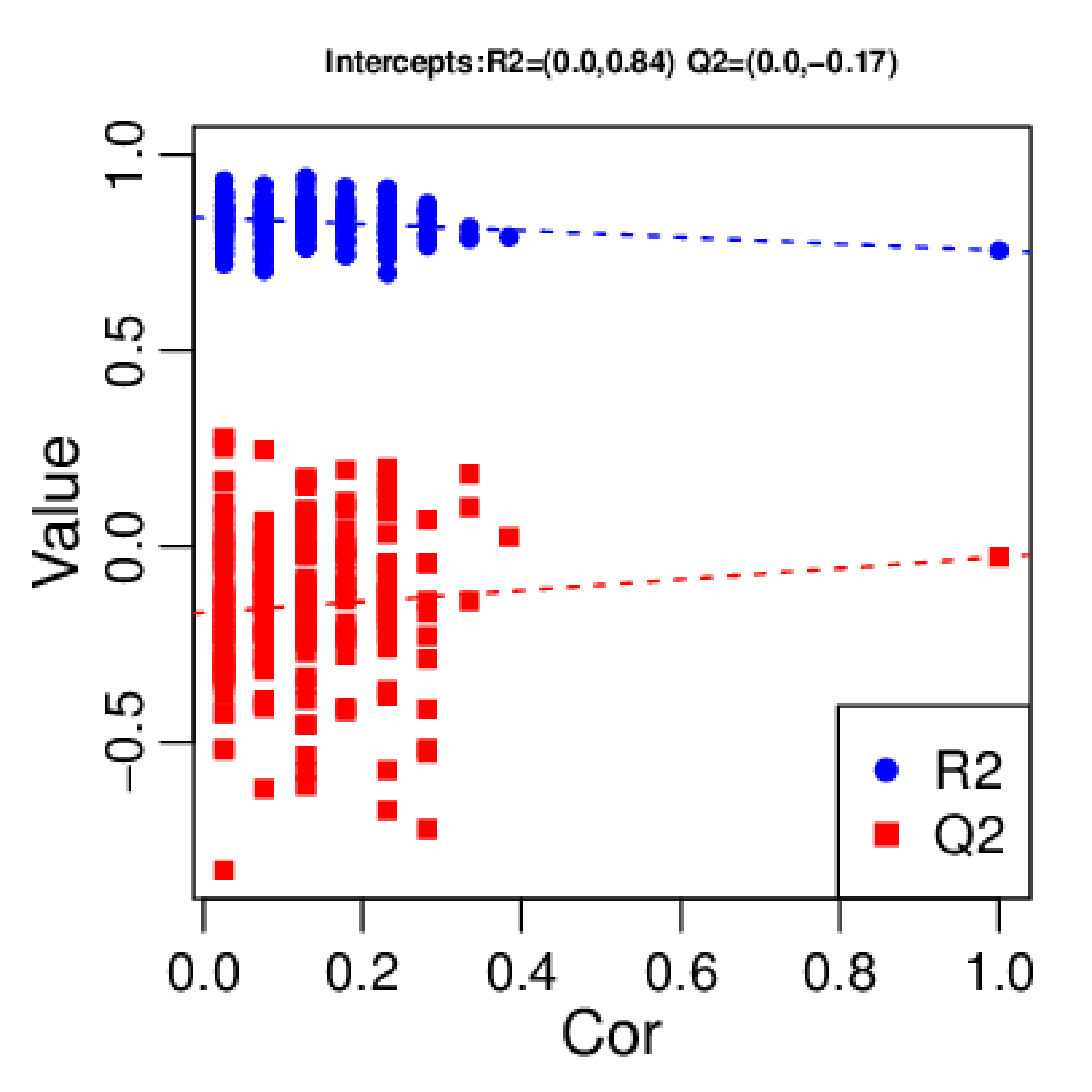

Supplement: Supplementary file 5 — Supplementary Material 5 [file 13018_2024_4713_MOESM5_ESM.jpg]
